# Supplementary material for: Large scale automated phylogenomic analysis of bacterial isolates and the Evergreen Online platform
Source: Commun Biol. 2020 Mar 20;3:137. doi: 10.1038/s42003-020-0869-5 (PMC7083913; doi:10.1038/s42003-020-0869-5)
Supplement: Supplementary file 1 — Supplementary Information [file 42003_2020_869_MOESM1_ESM.docx]

# Supplementary Information

## Supplementary Figures

*Supplementary Figure 1 Computational time of the Escherichia coli in vitro evolution dataset where the samples were added in batches based on the sampling time.*


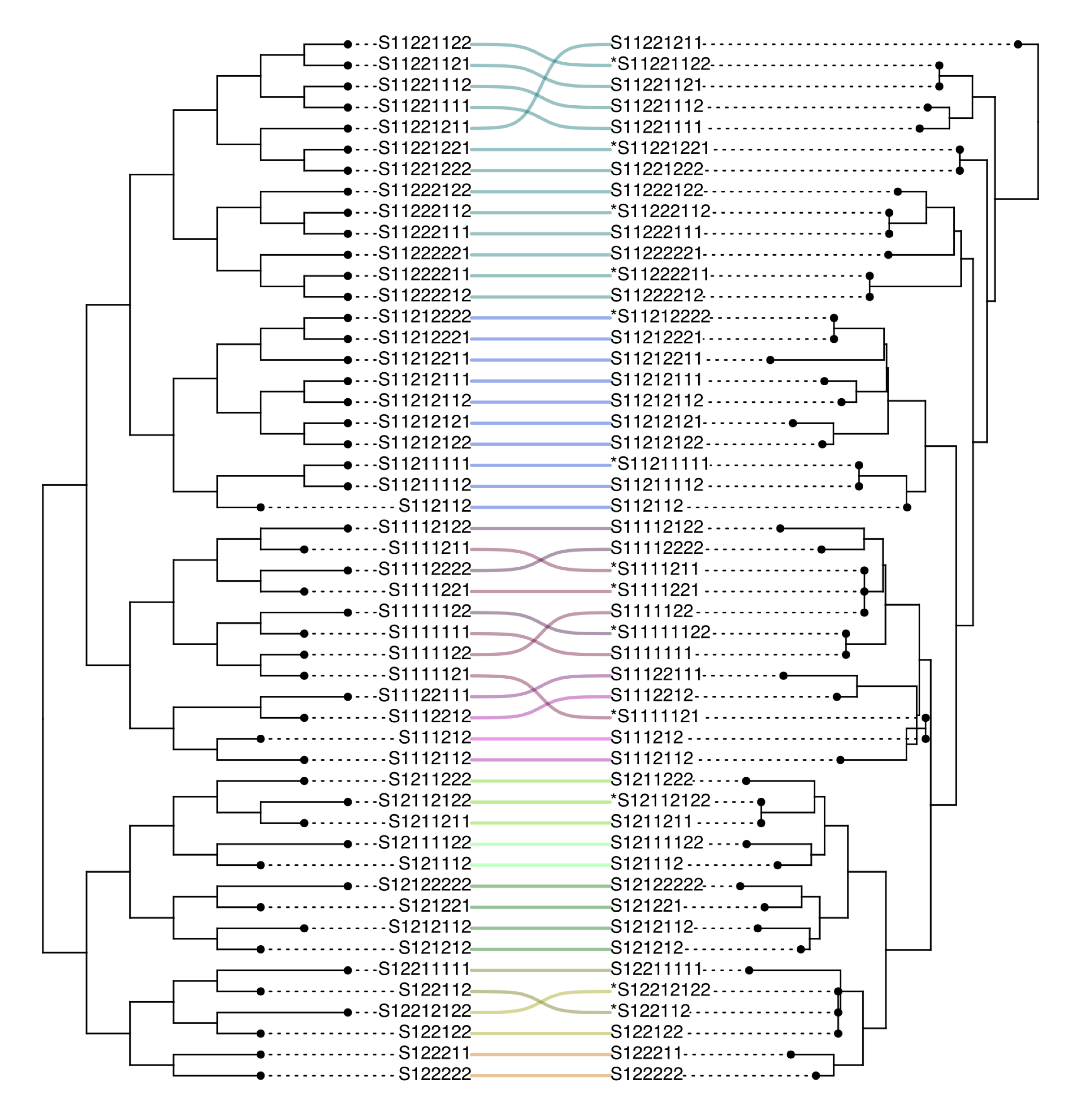


*Supplementary Figure 2 Comparison of the ideal tree to the PAPABAC neighbor-joining tree made of the in vitro experiment dataset* [1] *Taxa with an asterisk were clustered together with the taxa in the same clade.*

*Supplementary Figure 3 Maximum likelihood trees of (A-B) Campylobacter jejuni and (C-D) Listeria monocytogenes SNP pipeline benchmarking datasets. The trees A,C are the “ideal” phylogenies by Timme et al.* [2] *The colored (blue, red) clades contain the outbreak strains, while the black ones are non-related isolates. The reference sequences were trimmed from the trees.*

*Supplementary Figure 4 A) Number of downloaded and included isolates as function of data acquisition events B) Number of isolates for the species we query for C) Fraction of non-redundant isolates in template sets larger than 100 isolates*


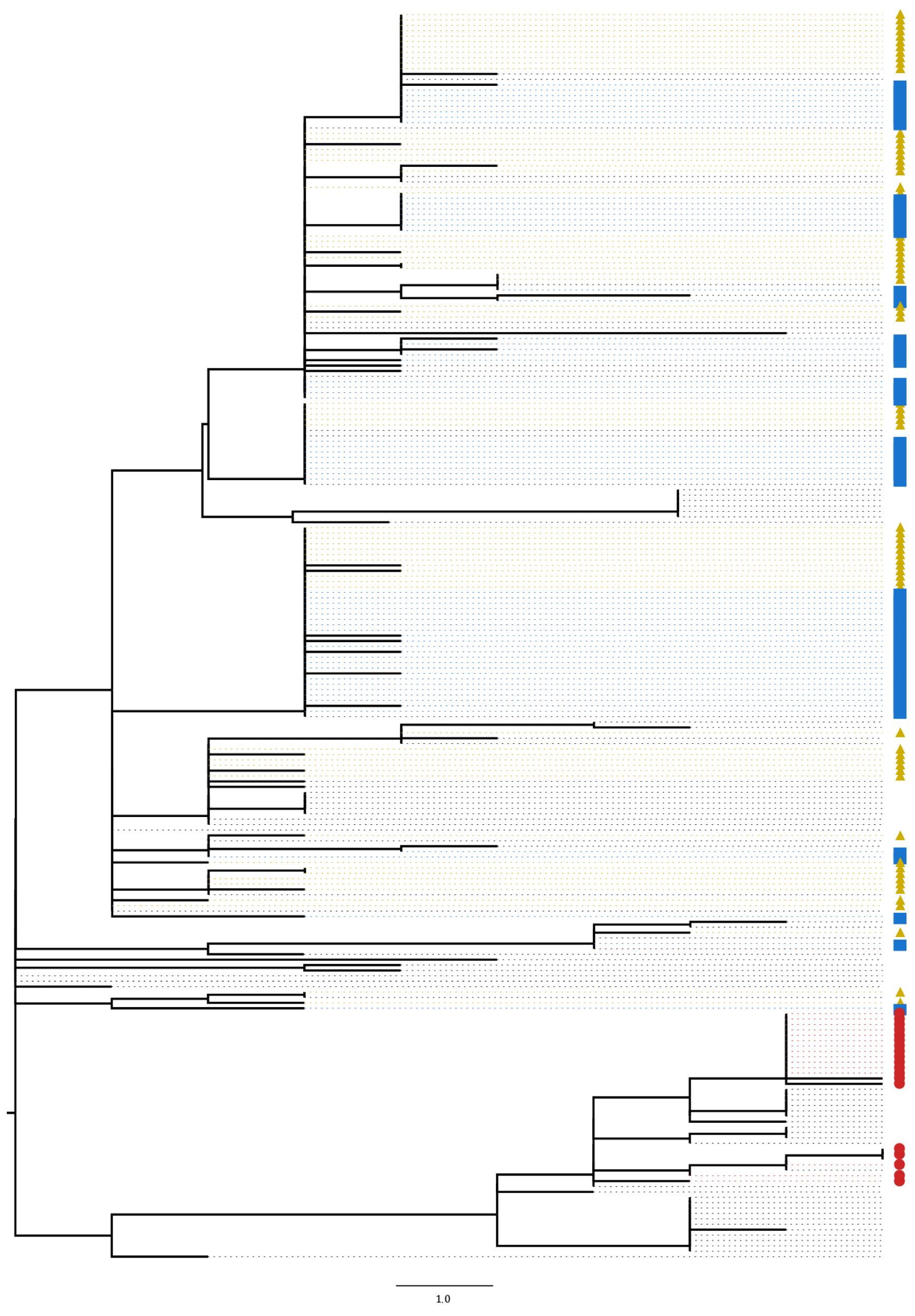


*Supplementary Figure 5: The refined subtree around isolate SRR6766978 as of 30.10.2019, pruned to contain the same isolates as Figure 5. Labeled samples are the same as Figure 5.*

## Supplementary Tables

*Supplementary Table 1 Non-queried species, due to mislabeled or mixed samples as of 2018.06.27*

| Genus | Species | Isolate |
| --- | --- | --- |
| *Bacillus* | *subtilis* | 3 |
| *Bacillus* | *pumilus* | 2 |
| *Campylobacter* | *coli* | 58 |
| *Campylobacter* | *fetus* | 1 |
| *Citrobacter* | *amalonaticus* | 1 |
| *Enterobacter* | *cloacae* | 2 |
| *Enterococcus* | *faecalis* | 1 |
| *Escherichia* | *albertii* | 5 |
| *Hafnia* | *alvei* | 3 |
| *Klebsiella* | *pneumoniae* | 7 |
| *Listeria* | *ivanovii* | 1 |
| *Morganella* | *morganii* | 7 |
| *Peptoclostridium* | *difficile* | 1 |
| *Proteus* | *mirabilis* | 7 |
| *Providencia* | *stuartii* | 2 |
| *Pseudomonas* | *aeruginosa* | 6 |
| *Raoultella* | *ornithinolytica* | 1 |
| *Salmonella* | *bongori* | 11 |
| *Staphylococcus* | *epidermidis* | 1 |
| *Streptococcus* | *agalactiae* | 1 |

## Supplementary References

[1] J. Ahrenfeldt, C. Skaarup, H. Hasman, A. G. Pedersen, F. M. Aarestrup, and O. Lund, “Bacterial whole genome-based phylogeny: construction of a new benchmarking dataset and assessment of some existing methods,” *BMC Genomics*, vol. 18, no. 1, p. 19, Dec. 2017.

[2] R. E. Timme *et al.*, “Benchmark datasets for phylogenomic pipeline validation, applications for foodborne pathogen surveillance,” *PeerJ*, vol. 5, p. e3893, Oct. 2017.
