# Supplementary material for: Large scale automated phylogenomic analysis of bacterial isolates and the Evergreen Online platform
Source: Commun Biol. 2020 Mar 20;3:137. doi: 10.1038/s42003-020-0869-5 (PMC7083913; doi:10.1038/s42003-020-0869-5)
Supplement: Supplementary file 4 — Description of Additional Supplementary Files [file 42003_2020_869_MOESM4_ESM.pdf]

Description of Supplementary Data

File Name: Supplementary Data 1
Description: Data parsed from logs of wrapper 2 of Evergreen Online until 2018-08-31

File Name: Supplementary Data 2
Description: Common samples between NCBI Pathogen Detection platform tree PDS000000952.271 and Evergreen Online phylogenetic tree
